# The 4Ms Provide A Structural Framework For Organizing Educational Materials

**DOI:** 10.1093/geroni/igab046.2804

**Published:** 2021-12-17

**Authors:** Edmund Duthie, Deborah Simpson, Amanda Szymkowski, Kathryn Denson, Steven Denson

**Affiliations:** 1 Medical College of Wisconsin, Milwaukee, Wisconsin, United States; 2 Advocate Aurora Health, Advocate Aurora Health/Milwaukee, Wisconsin, United States; 3 Medical College of Wisconsin, Medical College of Wisconsin/Milwaukee, Wisconsin, United States

## Abstract

The John A Hartford Foundation and the Institute for Healthcare Improvement (IHI)’s 4Ms of mentation, mobility, medications and (what) matters most provide a much-needed framework for helping system leaders and frontline teams consistently deliver high-quality, age-friendly care. Geriatric Fast Facts (GFFs) is a virtual resource providing teachers/learners with peer-reviewed, evidence-based summaries on topics essential to older adult care via a searchable website [www.geriatricfastfacts.com]. To determine if GFFs can be classified by the 4Ms we initially did a free text search of all GFFs. That revealed GFFs whose foci were unrelated to the 4Ms (e.g., mobility emerged in a fluoroscopy GFF as a minor element related to patient positioning). Therefore, all GFFs were independently reviewed by a geriatrician and the website manager and classified according to the 4M rubric (a single GFF can be classified in multiple M’s such as #93 on Age Friendly Health Systems). Any differences were adjudicated by the GFF editor. 64% (60/ 93) of GFFs strongly linked to one of the 4Ms. The number of GFFs dedicated to the 4Ms are as follows: 20 what matters most, 18 medications, 13 mentation, and 9 mobility. Those that were not coded within 4Ms were often very disease/specialty oriented. A total of 36 were not classified. For example, GGF #39 focuses on the etiologies of anemia among older adults. The 4M framework can be easily applied to educational materials to support consistent and clear conceptual model across learning conditions and materials.

